# Genetic Modification of the Effects of Exercise Behavior on Mental Health

**DOI:** 10.3389/fpsyt.2014.00064

**Published:** 2014-06-03

**Authors:** Nienke M. Schutte, Meike Bartels, Eco J. C. de Geus

**Affiliations:** ^1^Department of Biological Psychology, Faculty of Psychology and Education, VU University Amsterdam, Amsterdam, Netherlands; ^2^EMGO+ Institute for Health and Care Research, VU University Medical Center Amsterdam, Amsterdam, Netherlands

**Keywords:** exercise, depression, wellbeing, mood effects, individual differences, genetics

Anxiety and depressive disorders are a major contributor to the global disease burden (1). Although these disorders differ in duration and intensity, they are often chronic and treatment options include medication, psychotherapy, or a combination of both. In addition, regular exercise is argued to be effective in reducing anxious and depressive symptoms. Results from several meta-analyses indicate that exercise has a moderate to large antidepressant effect in clinical populations (2–6). Based on these studies, one might easily conclude that exercise consistently has beneficial causal effects on anxious and depressive symptoms (7).

The question remains whether this conclusion is also valid with regard to the general population as, despite these beneficial psychological effects, the majority of the population is not engaging in leisure-time exercise activities (8, 9) and population studies on the association between exercise and mental health are scarce. Secondly, there may be mechanisms that only mimic causal effects. The observed association between exercise and anxious–depressive symptoms might be due to underlying factors that influence both exercise behavior and symptoms of anxiety and depression. These factors can reside in the environment or in our genes. Underlying genetic factors might for instance have a detrimental effect on regular exercise behavior while simultaneously increasing the risk for depression, a mechanism known as genetic pleiotropy. The effect of these genetic factors on exercise behavior could even precede their effects on depression, thereby nearly perfectly mimicking a causal association. Only a few research groups have the optimal resources to investigate these possible effects in a genetically informative design, which requires large population-based longitudinal datasets with family data, but preferably twin data.

Results from population-based twin studies that have tested the nature of the association between a lack of exercise and anxious–depressive symptoms conclude that the association is best explained by underlying genetic effects. De Moor et al. (10) showed that within genetically identical twins, a twin who exercised more did not have fewer symptoms than his or her less exercising co-twin. This suggests that genetic factors independently cause low levels of exercise behavior as well as anxious and depressive symptoms. In addition, there is no evidence for causal influences of exercise behavior on feelings of psychological wellbeing, a phenotype presumably at the other end of the emotional scale, i.e., the absence of anxious or depressive symptoms (11, 12). Taken together, these studies conclude that the association between regular exercise and psychological wellbeing as well as the association between a lack of regular exercise and anxiety and depressive disorders largely reflect the effects of common genetic factors.

In an effort to explain the mechanisms that contribute to the association between exercise activities and mental health in the general population, a model was proposed that accommodates genetic pleiotropic effects, but still allows exercise to causally increase wellbeing in specific subgroups of the population (13). As with any other behavior, for exercise behavior to be repeated regularly, the net appetitive effects of exercise would need to outweigh the net aversive effects. Individuals who experience greater exercise induced mood enhancement are likely to repeat the behavior and become regular lifetime exercisers. This assumption is supported by several studies, which show that a more positive affective response during exercise was associated with greater participation in (voluntary) moderate to vigorous exercise (14, 15) or the intention to engage in voluntary exercise (16). Individual differences in these acute mood effects of exercise could be strongly co-determined by genetic factors.

In addition to differential acute mood effects, there could be a social-psychological mechanism that makes some individuals more attracted to exercise than others. Individuals with higher innate exercise capacities will gain more in exercise performance than others at comparable levels of training. The higher trainability and the superior exercise performance will lead to feelings of competence and mastery. This increased confidence, or self-efficacy, may not only enhance the frequency of exercise in individuals (17), but will also lead to higher self-esteem and in turn, in feelings of wellbeing. Vice versa, low trainability and lower levels of performance will lead to disappointment and particularly in adolescents to shame and lowered self-esteem. Genetic variation among people influencing exercise ability will therefore become associated with experiencing psychological beneficial effects of exercise activities and, as a consequence, with an increase in the frequency of exercising.

Major future challenges are to test the association between the level of voluntary exercise behavior and the acute and longer term psychological responses to exercise, and to establish the contribution of shared genetic factors to these associations. This requires a substantial family or twin study with measurements of exercise ability and the acute mood response to exercise. Various experimental design issues should be taken into account in these studies. First, the intensity at which an individual is exercising is an important determinant of the aversive responses to exercise: at intensities that exceed the individuals’ ventilatory threshold (VT), when there is a transition from aerobic to anaerobic metabolism, negative changes in exercise induced mood response are observed (18). Measurements should therefore be standardized for the VT. Second, different types of exercise induced mood responses can be measured: during (immediate response on exercising) or (shortly) after the exercise bout (more complex, long lasting feelings). These responses may differ in origin, but are likely to contribute to the overall balance of appetitive and aversive effects of exercise, therefore, should both be included in measurements. For the assessment of exercise ability it is important to take into account a range of objective determinants like aerobic fitness, balance, flexibility and static, and dynamic muscle strength, but also record self-perceived exercise ability, particularly in relation to the relevant peer group.

Acknowledgment of the differential sensitivity to the psychological effects of exercise is of great importance. Some individuals may require a specific exercise program (with respect to intensity of exercise, absence or presence of competitive elements, and type of exercise) to create a situation in which the appetitive effects of exercise can predominate. This may ensure that these individuals continue to be engaged in regular exercise while maximizing their psychological benefits in terms of increased feelings of wellbeing and decreased levels of anxiety and depression.

## Conflict of Interest Statement

The authors declare that the research was conducted in the absence of any commercial or financial relationships that could be construed as a potential conflict of interest.
